# 中国真实世界慢性髓性白血病患者停用酪氨酸激酶抑制剂的观察研究

**DOI:** 10.3760/cma.j.issn.0253-2727.2022.08.004

**Published:** 2022-08

**Authors:** 慧芳 赵, 云帆 杨, 兵城 刘, 纬明 黎, 娜 许, 晓力 刘, 倩 江, 惠兵 党, 立新 梁, 䶮莉 张, 永平 宋

**Affiliations:** 1 河南省肿瘤医院，郑州大学附属肿瘤医院血液科，郑州 450008 Department of Hematology, Henan Cancer Hospital, the Affiliated Cancer Hospital of Zhengzhou University, Zhengzhou 450008, China; 2 四川大学华西医院血液内科，血液病研究所，成都 610041 Department of Hematology, Institute of Hematology, West China Hospital, Sichuan University, Chengdu 610041, China; 3 中国医学科学院血液病医院（中国医学科学院血液学研究所），天津 300020 Institute of Hematology & Blood Diseases Hospital, Chinese Academy of Medical Sciences & Peking Union Medical College, Tianjin 300020, China; 4 华中科技大学同济医学院附属协和医院血液科，武汉 430022 Department of Hematology, Union Hospital, Tongji Medical College, Huazhong University of Science and Technology, Wuhan 430022, China; 5 南方医科大学南方医院血液科，广州 510515 Department of Hematology, Nanfang Hospital, Southern Medical University, Guangzhou 510515, China; 6 北京大学人民医院、北京大学血液病研究所、国家血液系统疾病临床医学研究中心，北京 100044 Peking University People's Hospital, Peking University Institute of Hematology, National Clinical Research Center for Blood System Diseases, Beijing 100044, China; 7 南阳医学高等专科学校第一附属医院血液科，南阳 473000 The First Affiliated Hospital of Nanyang Medical College, Nanyang 473000, China; 8 三门峡市中心医院血液科，三门峡 472000 Department of Hematology, Sanmenxia Central Hospital, Sanmenxia 472000, China

**Keywords:** 停药, 二代酪氨酸激酶抑制剂, 伊马替尼, 白血病，髓样，慢性, 无治疗缓解, Deprescriptions, Second-generation tyrosine kinase inhibitors, Imatinib, Leukemia, myeloid, chronic, Treatment free remission

## Abstract

**目的:**

观察真实世界中酪氨酸激酶抑制剂（TKI）治疗慢性髓性白血病（CML）停药患者的无治疗缓解（treatment free remission, TFR）情况，及二代TKI在TFR方面是否优于伊马替尼（IM）。

**方法:**

对来自国内8家血液病治疗中心的自2013年6月至2021年3月期间停用TKI且有明确停药结局和相对完整临床资料的274例CML患者进行回顾性分析，使用单因素分析和多因素Cox比例风险回归模型评估影响CML患者停药后TFR结局的危险因素。

**结果:**

274例患者，女性140例（51.1％），停药时中位年龄48（9～84）岁。停药前，172例（62.8％）患者应用IM治疗，102例（37.2％）接受过二代TKI治疗，包括73例IM转换二代TKI者和29例一线二代TKI治疗者。转换二代TKI的原因包括：37例因追求TFR在IM治疗获得深层分子学反应（DMR）期间转换二代TKI、7例因IM不良反应不耐受、29例因TKI治疗未达最佳治疗反应。接受过二代TKI者末次应用TKI类型包括：尼洛替尼96例（94.1％），达沙替尼3例（2.9％），氟马替尼2例（2.0％），1例为二代TKI不耐受再次更换为IM治疗。IM治疗组和二代TKI治疗组在确诊时中位年龄、停药时中位年龄、性别、Sokal评分、TKI治疗前是否应用干扰素、TKI治疗至获得DMR中位时间、停药原因等基线特征差异均无统计学意义（*P*值均>0.05）。停药前TKI中位累积治疗时间（71.5个月对88个月，*P*<0.001）、末次TKI中位治疗时间（60个月对88个月，*P*<0.001）、DMR中位持续时间（58个月对66个月，*P*＝0.002），二代TKI治疗组均较IM治疗组显著缩短。停药后中位随访22（6～118）个月，88例（32.1％）患者在中位6（1～91）个月失去主要分子学反应（MMR），其中53例（60.2％）患者在≤6个月内失去MMR，总体的TFR率67.9％，12个月和24个月的累积TFR率分别为70.5％和67.5％。26例（9.5％）患者发生停药综合征。72例（83.7％）重启TKI治疗患者在中位治疗4（1～18）个月再次获得DMR。单因素分析结果显示，停药前应用二代TKI治疗组患者的TFR率显著高于IM治疗组（77.5％对62.2％，*P*＝0.041）。进一步亚组分析发现，IM转换二代TKI治疗组患者的TFR率显著高于IM治疗组（80.8％对62.2％，*P*＝0.026），二代TKI一线治疗组和IM治疗组TFR率差异无统计学意义（69.0％对62.2％，*P*＝0.599）。多因素分析结果显示，应用二代TKI治疗是影响停药患者TFR的独立预后因素（*RR*＝1.827，95％*CI* 1.015～3.288，*P*＝0.044）。

**结论:**

真实世界中，二代TKI治疗较IM治疗使更多的CML患者更早获得更好的TFR。

## Abstract (en)

**Objective:**

This study aimed to observe whether the treatment-free remission（TFR）of second-generation tyrosine kinase inhibitors（TKI）in chronic myeloid leukemia（CML）is better than imatinib（IM）.

**Methods:**

The clinical data of 274 CML patients who discontinued treatment and with complete clinical data were retrospectively studied from June 2013 to March 2021. Using both univariate and multivariate Cox proportional hazards regression models, risk factors influencing TFR outcomes after drug withdrawal in CML patients were assessed.

**Results:**

A total of 274 patients were enrolled, 140 patients were women（51.1％）, with a median age of 48（9–84）years at the time of TKI discontinuation. Prior to TKI discontinuation, 172（62.8％）patients were treated with IM, and 102（37.2％）had received second-generation TKI treatment, including 73 patients who had shifted from IM to a second-generation TKI and 29 patients who used second-generation TKI as the first-line treatment. The rationale for converting to a second-generation TKI are as follows: 37 patients aimed deep molecular response（DMR）to achieve TFR, seven patients changed due to IM intolerance, and 29 patients changed because of failure to achieve the optimal treatment response. The use of the last type of TKI included 96 patients（94.1％）with nilotinib, three patients（2.9％）with dasatinib, and two patients（2％）with flumatinib, including one patient who changed to IM due to second-generation TKI intolerance. No statistical differences were found in the median age at diagnosis and TKI discontinuation, sex, Sokal score, IFN treatment before TKI, median time of TKI treatment to achieve DMR, and the reasons for TKI discontinuation between the second TKI and IM（*P*>0.05）.The median cumulative treatment time of TKI（71.5 months *vs* 88 months, *P*<0.001）, the last TKI median treatment time（60 months *vs* 88 months, *P*<0.001）, and the median duration of DMR（58 months *vs* 66 months, *P*＝0.002）were significantly shorter in the second-generation TKI compared with IM. In the median follow-up of 22（6–118）months after TKI discontinuation, 88 patients（32.1％）had lost their MMR at a median of 6（1–91）months; of the 53 patients（60.2％）who lost MMR within 6 months, the overall TFR rate was 67.9％, and the cumulative TFR rates at 12 and 24 months were 70.5％ and 67.5％, respectively. Withdrawal syndrome occurred in 26 patients（9.5％）. For patients who restarted TKI treatment, 72 patients（83.7％）achieved DMR again at a median treatment of 4（1 to 18）months. The univariate analysis showed that the TFR rate of patients treated with second-generation TKI was significantly higher than those who were treated with IM（77.5％ *vs* 62.2％, *P*＝0.041）. A further subgroup analysis found that the TFR rate of the second-generation TKI patients was significantly higher than those treated with IM（80.8％ *vs* 62.2％, *P*＝0.026）. No significant difference was found in the second-generation TKI used as the first line treatment compared with those who were treated with IM（69.0％ *vs* 62.2％, *P*＝0.599）. The multivariate analysis results showed that second-generation TKI treatment was an independent prognostic factor affecting TFR in patients who discontinued TKI（*RR*＝1.827, 95％*CI* 1.015–3.288, *P*＝0.044）.

**Conclusion:**

In the clinical setting, more CML patients rapidly achieved TFR using second-generation TKI than IM treatment.

一代和二代酪氨酸激酶抑制剂（TKI）的应用，极大地改善了慢性髓性白血病（CML）患者的预后，CML患者的预期寿命已接近正常同龄人群[1]。当长期生存不是问题，改善生活质量和追求无治疗缓解（treatment free remission, TFR）逐渐成为CML患者治疗的新目标。而获得深层分子学反应（DMR）是CML患者追求TFR的前提。多中心、大样本、随机对照研究结果显示，二代TKI尼洛替尼（NIL）、达沙替尼（DAS）可较伊马替尼（IM）使CML患者更快获得更深层的分子学反应[2]–[5]。那么在获得TFR方面二代TKI治疗是否仍较IM治疗更有优势？目前国内尚缺乏多中心、大样本研究数据。本研究对来自国内8家血液病治疗中心的274例停药CML患者的临床资料进行回顾性分析，现报道如下。

## 病例与方法

1. 病例资料：回顾性收集来自国内8家血液病治疗中心自2013年6月至2021年3月期间停用TKI且随访≥6个月的CML患者的资料，将其中停药前获得持续稳定DMR≥2年且具有明确停药结局及临床资料相对完整的274例患者纳入研究。

纳入标准：①服用TKI前确诊BCR-ABL P210型阳性的CML慢性期（CP）或加速期（AP）（ELN分期）患者；②在规范的国际标准化检测下，TKI治疗反应达持续稳定DMR≥2年且有强烈停药意愿和（或）不良反应和（或）合并症的患者；③无TKI耐药史的患者；④停药后能够坚持定期检测者。

排除标准：①既往接受造血干细胞移植或细胞免疫治疗的患者；②TKI治疗期间Ph阴性染色体间期出现克隆性染色体异常者；③有急变史的患者；④BCR-ABL转录本为非P210型患者；⑤停药前TKI治疗不规范且未获得持续稳定DMR的患者；⑥合并第二肿瘤的患者。

2. 疗效评估标准和停药后疾病监测频率：疗效评估标准参照《中国慢性髓性白血病诊疗监测规范（2016年版）》[6]。DMR定义为MR^4^（BCR-ABL^IS^≤0.01％，ABL转录本>10 000）或MR^4.5^（BCR-ABL^IS^≤0.0032％，ABL转录本>32 000）。停药后疾病监测频率：前6个月每月行1次分子学检测，第6～12个月每1～2个月复查1次，12个月后每3～6个月复查1次。失去主要分子学反应（MMR）后，应尽快重启TKI治疗。

3. TFR和停药综合征的定义：TFR定义为自停用TKI至丧失MMR、任何原因导致的死亡、再次服用TKI时结束。停药综合征指停用TKI后数周至数月内出现短暂的肌肉骨骼疼痛。

4. 随访：通过查看门诊记录及电话咨询的方式随访。自停药开始随访，随访截至2021年6月30日。

5. 统计学处理：采用IBM SPSS 25.0软件进行统计学分析。描述性方法分析患者人口学和临床特征。定量变量比较采用Mann-Whitney *U*法检验，分类变量比较采用卡方检验或Fisher精确检验。采用Kaplan-Meier评估TFR率，采用Log-rank检验进行单因素组间TFR差异分析，采用Cox比例风险回归模型对影响TFR的多个因素进行分析。*P*<0.05为差异有统计学意义。

## 结果

一、患者基线特征

274例患者，女140例（51.1％），男134例（48.9％），停药时中位年龄48（9～84）岁。全部患者确诊时均处于CP，其中5例（1.8％）患者应用TKI治疗前有AP病史（经TKI治疗后获得持续稳定DMR）。182例（66.4％）患者确诊时具有明确Sokal评分，包括低危100例（36.5％）、中危58例（21.2％）、高危24例（8.8％）。1例患者确诊时染色体为复杂核型、1例为变异染色体。15例（5.5％）患者在接受TKI治疗之前曾接受干扰素（IFN-α）治疗，5例（1.8％）患者在接受TKI治疗之前曾接受化疗。停药原因分别为：TKI治疗获得持续稳定DMR 155例（56.6％）、药物不良反应63例（23％）、计划或意外妊娠31例（11.3％）、经济原因22例（8％）、其他3例（1.1％）。

274例患者，停药前所有类型TKI中位累积治疗 81（36～222）个月，TKI治疗至获得DMR的中位时间12（2～140）个月，停药前DMR中位持续时间62（24～153）个月。停药前172例（62.8％）患者持续应用IM治疗，102例（37.2％）接受过二代TKI治疗（二代TKI治疗组），包括73例IM转换二代TKI者和29例一线二代TKI治疗者，其中转换二代TKI的原因：37例因追求TFR在IM治疗获得DMR期间转换二代TKI、7例因IM不良反应不耐受、29例因TKI治疗未达最佳治疗反应（TKI疗效“警告”，无患者检测到ABL激酶区突变）。二代TKI治疗组患者，末次应用TKI类型包括：NIL 96例（94.1％），DAS 3例（2.9％），氟马替尼（FM）2例（2％），1例为二代TKI不耐受再次更换为IM治疗。IM治疗组和二代TKI治疗组患者在确诊时中位年龄、停药时中位年龄、性别、Sokal评分、TKI治疗前是否应用IFN、TKI治疗至获得DMR中位时间、停药原因等基线特征差异均无统计学意义（*P*值均>0.05）。而停药前TKI中位累积治疗时间（71.5个月对88个月，*P*<0.001）、停药前末次TKI中位治疗时间（60个月对88个月，*P*<0.001）以及停药前DMR中位持续时间（58个月对66个月，*P*＝0.002），二代TKI治疗组均较IM治疗组明显缩短。274例停药患者的基线特征见表1。

**表1:** 274例慢性髓性白血病患者的基线特征

临床特征	患者总体（274例）	IM组（172例）	二代TKI组（102例）	统计量	*P*值
确诊时年龄［岁，*M*（范围）］	40（3～69）	39（3～67）	42（6～69）	-1.007	0.314
停药时年龄［岁，*M*（范围）］	48（9～84）	47（9～82）	49（11～84）	-0.692	0.489
女性［例（％）］	140（51.0）	85（49.4）	55（53.9）	0.520	0.471
Sokal评分［例（％）］				1.248	0.536
低危	100（36.4）	64（37.2）	36（35.2）		
中危	58（21.1）	32（18.6）	26（25.4）		
高危	24（8.7）	14（8.1）	10（9.8）		
未知	87（31.7）	60（34.8）	27（26.4）		
TKI治疗前应用IFN治疗［例（％）］	15（5.5）	12（7.0）	3（2.9）	2.015	0.156
停药前末次应用TKI类型［例（％）］				269.745	<0.001
IM	173（63.1）	172（100）	1（1.0）		
NIL	96（35.0）	0（0）	96（94.1）		
DAS	3（1.1）	0（0）	3（2.9）		
FM	2（0.7）	0（0）	2（2.0）		
停药前TKI累积治疗时间［月，*M*（范围）］	81（36～222）	88（36～172）	71.5（39～222）	-4.844	<0.001
停药前末次TKI治疗持续时间［月，*M*（范围）］	72（5～172）	88（36～172）	60（5～97）	-10.234	<0.001
初始TKI治疗至获得DMR时间［月，*M*（范围）］	12（2～140）	13（2～140）	12（3～112）	-1.795	0.073
停药前DMR持续时间［月，*M*（范围）］	62（24～153）	66（24～153）	58（24～141）	-3.088	0.002
停药原因［例（％）］				8.662	0.070
持续DMR	155（56.6）	95（55.2）	60（58.8）		
药物不良反应	63（23.0）	40（23.3）	23（22.5）		
妊娠	31（11.3）	19（11.0）	12（11.8）		
经济困难	22（8.0）	18（10.5）	4（3.9）		
其他	3（1.1）	0（0）	3（2.9）		
停药后发生骨痛［例（％）］	26（9.5）	18（10.5）	8（7.8）	0.513	0.474
TFR［例（％）］	186（67.9）	107（62.2）	79（77.5）	6.823	0.009

注：IM：伊马替尼；TKI：酪氨酸激酶抑制剂；IFN：干扰素；NIL：尼洛替尼；DAS：达沙替尼；FM：氟马替尼；DMR：深层分子学反应；TFR：无治疗缓解

二、停药患者TFR结局和再治疗转归

274例患者，停药后中位随访22（6～118）个月，88例（32.1％）患者在停药后中位6（1～91）个月失去MMR，总的TFR率67.9％，其中53例（60.2％）患者在≤6个月内失去MMR，21例（23.9％）在6～12个月失去MMR，14例（15.9％）在>12个月失去MMR，其中9例（10.2％）在停药24个月后失去MMR。12个月和24个月的累积TFR率分别为70.5％和67.5％。

88例失去TFR的患者，其中86例重启TKI治疗，再治疗TKI类型包括：IM 58例（67.4％），NIL 20例（23.3％），DAS 8例（9.3％）。至随访结束，72例（83.7％）中位治疗4（1～18）个月再次获得DMR；无患者进展为AP或急变期（BC），仅1例停药1个月后失去MMR的27岁男性，检出V299L突变，更换NIL治疗第14个月重获DMR。

三、停药安全性

至随访结束，274例患者停药期间，无患者发生严重不良事件。停药后，26例（9.5％）患者发生停药综合征，骨痛程度大多为1～2级，经口服解热镇痛药物可缓解。IM治疗组和二代TKI治疗组患者停药综合征的发生率差异无统计学意义（10.5％对7.8％，*P*＝0.474）。

四、影响TFR的相关因素分析

1. 单因素分析：将性别、年龄、Sokal评分、是否应用过IFN、TKI治疗至获得DMR时间，停药前TKI治疗时间、TKI治疗类型、是否应用过二代TKI、DMR持续时间等纳入单因素分析。性别、年龄、Sokal评分、是否应用IFN、TKI治疗至获得DMR时间、停药前TKI治疗持续时间、停药前DMR持续时间等因素对TFR无明显影响（*P*值均>0.05）。仅发现应用二代TKI治疗组TFR率明显升高（77.5％对62.2％，*P*＝0.041）（表2、图1）。

**表2:** 影响慢性髓性白血病患者无治疗缓解（TFR）的单因素分析

因素	例数	TFR率（％）	*χ*^2^值	*P*值
停药时年龄			2.267	0.132
≥48岁	143	72.0		
<48岁	131	63.4		
性别			0.510	0.475
男	134	64.9		
女	140	70.7		
Sokal评分			0.398	0.819
低危	100	66.0		
中危	58	72.4		
高危	24	62.5		
是否应用IFN			0.677	0.411
是	15	80.0		
否	256	67.2		
TKI治疗持续时间			0.376	0.540
≥80个月	150	68.0		
<80个月	124	67.7		
是否应用二代TKI			4.165	0.041
是	102	77.5		
否	172	62.2		
停药前TKI治疗类型				
IM一线治疗	172	62.2		
IM转换二代TKI治疗	73	80.8	4.954^a^	0.026^a^
二代TKI一线治疗	29	69.0	0.276^a^ 1.225^b^	0.599^a^ 0.268^b^
初始TKI治疗至获得DMR时间			1.412	0.235
≤12个月	128	72.7		
>12个月	106	63.2		
DMR持续时间			0.245	0.621
≥60个月	131	71.8		
<60个月	104	63.5		

注：IFN：干扰素；TKI：酪氨酸激酶抑制剂；IM：伊马替尼；DMR：深层分子学反应；^a^与IM一线治疗组比较，^b^与IM转换二代TKI治疗组比较

**图1:**
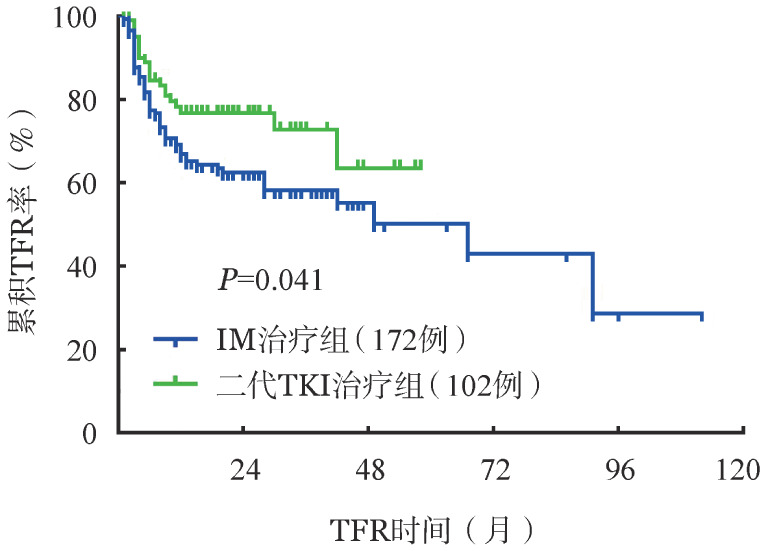
二代酪氨酸激酶抑制剂（TKI）治疗组和伊马替尼（IM）治疗组患者无治疗缓解（TFR）率的比较

进一步将患者分为IM治疗组（172例）、IM转换二代TKI组（73例）、二代TKI一线治疗组（29例）进行亚组分析。IM治疗组、IM转换二代TKI组和二代TKI一线治疗组患者停药前TKI中位治疗时间分别为88（41～172）、75（39～222）、60（42～97）个月，DMR持续中位时间分别为66（24～153）、58（24～141）、53.5（29～108）个月。发现IM转换二代TKI组TFR率仍较IM治疗组显著升高（80.8％对62.2％，*P*＝0.026），二代TKI一线治疗组和IM治疗组TFR率差异无统计学意义（69.0％对62.2％，*P*＝0.599）。三组患者TFR曲线见图2。

**图2:**
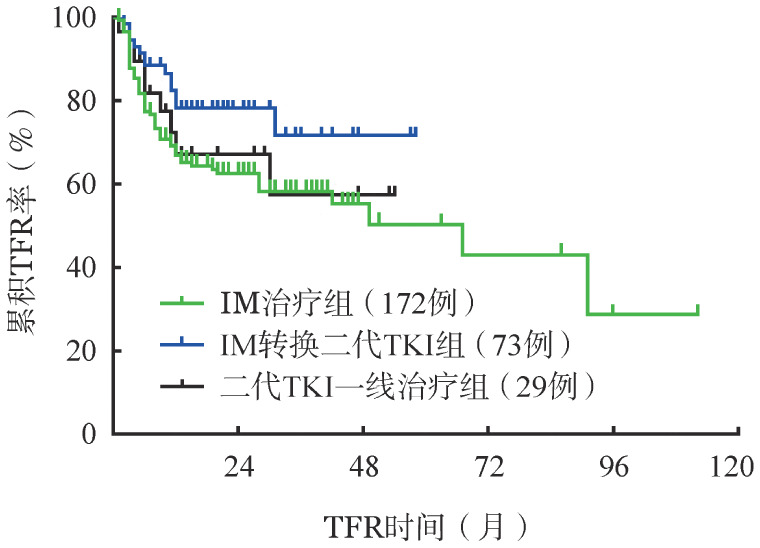
停药前应用不同类型酪氨酸激酶抑制剂（TKI）治疗组间无治疗缓解（TFR）率的比较 IM：伊马替尼

2. 多因素分析：将以上多个因素纳入Cox比例风险模型中进一步分析，结果显示：停药前应用过二代TKI治疗（*RR*＝1.827，95％*CI* 1.015～3.288，*P*＝0.044）是影响停药患者TFR的独立预后因素。

## 讨论

TKI的应用显著改善了CML患者的预后。NIL和IM治疗初诊CML的10年数据显示，NIL 300 mg和400 mg每日2次组、IM 400 mg/d组CML患者的总生存率分别为87.6％和90.3％、88.3％[4]–[5]。然而，随着TKI的长期使用，TKI相关不良事件、高额的治疗费用以及对育龄期女性生育的影响等问题逐渐凸显，影响CML患者的生活质量[4],[7]–[9]。国内一项多中心CML患者问卷调查结果显示，34％的患者由于生活质量低下愿意停用TKI[10]。本组274例患者停药原因依此为获得持续DMR 155例（56.6％）、药物不良反应63例（23％）、意外和计划妊娠31例（11.3％）、经济困难22例（8％）。

自STIM1研究[11]和TWISTER研究[12]结果相继发表以来，TFR研究逐渐成为CML领域研究的主要热点之一。而DMR几乎是所有TFR研究入组的关键指标[11],[13]–[23]。二代TKI NIL和DAS对比IM治疗初诊CML患者的多中心、随机对照研究结果显示，二代TKI的治疗应答率更高、应答更深、发生更早[2]–[3], [24]。DASISION研究的5年数据显示，DAS 100 mg/d治疗组5年累积MR^4.5^获得率42％，而IM 400 mg/d治疗组为33％[2]。ENESTnd研究5年更新的数据显示，NIL 300 mg每日2次治疗组累积MR^4.5^获得率54％，而IM 100 mg/d治疗组这一比例为31％[3]。德国CML-Study Ⅳ研究显示，若达到46％的MR^4.5^获得率，IM治疗需要8年[25]。Horn等[26]的研究结果发现，IM 400 mg/d治疗达MR^4^的中位时间为5.3～6.5年，而达到MR^4.5^的中位时间为9.1～10.7年。综上可见，二代TKI治疗可较IM治疗使更多的CML患者更快地达到追求TFR的“门槛”。

二代TKI治疗获得更高的DMR率能否转化为更高的TFR获益？目前尚未达成一致。将持续MR^4.5^≥2年设定为TFR入组条件，失去MMR定义为失去TFR的二代TKI和IM停药研究进行了纵向比较，在首个一线NIL治疗CML患者的TFR研究（ENESTfreedom研究）中，190例患者，12个月和48个月的TFR率分别为51.6％和49％[27]。在二线NIL（ENEStop研究）的长期TFR随访研究中，12个月和60个月的TFR率分别为58％和42.9％。另一项纳入60例一线或二线NIL或DAS治疗的CML患者的STOP2G-TKI研究中，12个月和48个月的TFR率分别为63.3％和53.7％。TRAD研究中，6个月TFR率为64.7％[28]。韩国的KID研究中90例IM治疗获得MR^4.5^≥2年的CML患者，12个月和24个月的TFR率分别为62.2％和58.5％[29]。从以上研究数据中似乎并未发现二代TKI治疗者的TFR率明显高于IM治疗者。然而，在一项旨在评估一线TKI治疗初诊CML-CP患者的DMR获得率和停药后分子无复发生存（MRFS）的研究[30]中，纳入了IM一线治疗者291例（73％），二代或三代TKI一线治疗者107例（27％），中位随访7年，182例（46％）患者获得了至少24个月的持续DMR，其中95例（24％）参加了TFR试验，12个月和48个月的MRFS率分别为55.1％和46.9％。多因素分析显示，二代或三代TKI一线治疗是影响停药后MRFS最显著的因素。Fava等[31]报道的一项意大利多中心停药研究显示，二代TKI治疗较IM治疗显示出更高的12个月的TFR率（73％对68％），且发现停药前二代TKI治疗组患者的TKI治疗持续时间（73个月对96个月，*P*＝0.002）和DMR持续时间均显著短于IM治疗组（36个月对53个月，*P*<0.001）。

本研究274例患者停药前62.8％的患者应用IM治疗，37.2％的患者接受过二代TKI治疗，中位随访22个月，总体TFR率为67.9％，基线特征和TFR率（62％）同Fava等[31]报道接近。本研究同样发现：二代TKI治疗组TFR率显著高于IM治疗组，且二代TKI治疗组患者停药前TKI中位累积治疗时间、末次TKI中位治疗时间、DMR中位持续时间均较IM治疗组显著缩短。在亚组分析中发现IM转换二代TKI组TFR率仍较IM治疗组显著升高（80.8％对62.2％，*P*＝0.026），但二代TKI一线治疗组较IM治疗组无显著升高（69％对62.2％，*P*＝0.599）。分析原因可能是：①在该研究中包含应用二代TKI一线治疗组的患者比例偏低，仅占总体人群的10.6％（29/274），可能会导致结果分析产生偏倚。②二代TKI一线治疗组患者停药前TKI治疗持续时间和DMR持续时间均较IM治疗组明显缩短。本研究中，IM治疗组和二代TKI一线治疗组患者停药前TKI中位治疗时间分别为88个月和60个月，停药前DMR中位持续时间分别为66个月和53.5个月。而停药前TKI治疗持续时间和DMR持续时间是影响停药患者TFR的重要因素[19]。综上，应用二代TKI治疗可较IM治疗使更多的CML患者更早获得更好的TFR。

停药综合征是临床医师和患者普遍关注的临床问题，本研究中，274例患者停药后，停药综合征总体发生率9.5％（26/274），骨痛程度大多为1～2级，经口服解热镇痛药物可缓解。两组患者骨痛的发生率差异无统计学意义（10.5％对7.8％）。IM治疗组停药综合征的发生率同KID研究[29]（15％）报道接近，但二代TKI停药组停药综合征的发生率远较Berger等[32]研究报道低。可能与不同TFR研究中停药前TKI治疗时间以及合并症情况存在差异相关。一项大型CML队列研究结果显示停药前有骨关节症状史（*RR*＝1.84）和TKI治疗时间长（*RR*＝1.68）是影响停药综合征发生的独立危险因素[32]。因此，CML患者在开始治疗前应仔细筛查，以确定是否存在骨关节症状，停药前应告知患者发生骨痛的可能性，尤其是在长期TKI治疗后停药的患者。

综上所述，本研究结果显示应用二代TKI治疗较IM治疗更有利于使CML患者更早获得更好的TFR，为真实世界期待追求TFR的CML患者的TKI治疗优化提供指导。本研究为回顾性研究，初诊时约33.6％的患者缺失Sokal评分以及本研究中IM转换二代TKI治疗的具体原因未进一步追溯，以上均可能导致分析结果的偏倚。更为精准的TFR数据仍有待进一步开展多中心、随机、对照研究。
